# Noncoplanar verification: a feasibility study using Philips' Pinnacle^3^ treatment planning system

**DOI:** 10.1120/jacmp.v16i6.5492

**Published:** 2015-11-08

**Authors:** Indra Yohannes, Heru Prasetio, Christoph Bert

**Affiliations:** ^1^ University Hospital Erlangen Radiation Oncology Erlangen Germany; ^2^ Friedrich‐Alexander‐Universitätsstr. Erlangen‐Nürnberg Faculty of Medicine Erlangen Germany; ^3^ Gesellschaft für Schwerionenforschung–Helmholtz Centre for Heavy Ion Research Department of Biophysics Darmstadt Germany

**Keywords:** noncoplanar, planar dose, Pinnacle^3^ TPS

## Abstract

Noncoplanar fields are normally used to improve the dose conformity of the target while sparing organs at risk. One of the methods to verify the dose distribution from the noncoplanar fields is by comparing their planar dose distributions from the treatment planning system (TPS) and the measured ones; for example, using film or electronic portal imaging devices (EPID). The planar dose distributions of the measurement tools, that are normally perpendicular to the central axis of the beam, can be calculated by creating special structures to mimic them in the TPS. With TPS commercially available today, however, it is not easy to create these special structures, especially in the noncoplanar configuration. For this work, we have written in‐house scripts in the Pinnacle^3^ TPS that can create the structures and define them as virtual planes. These virtual planes can be generated for any arbitrary gantry and couch angles, as well as source to planar distance, so that the planar dose maps at these planes can be computed. Two independent quality assurance (QA) tools were used to validate the planar dose distributions computed using the scripts for three open fields and one IMRT field at several different couch angles. The absolute planar dose patterns measured by the QA tools for all fields at all couch angles were found to be in good agreement, more than 95% (gamma criteria 3% delta dose and 3 mm distance to agreement), with the calculated ones by TPS. The results of this feasibility study can be valuable either for pretreatment dose verification or for *in vivo* dosimetry that directly implements the planar dose distributions from the TPS, particularly for the noncoplanar fields.

PACS numbers: 87.55.de, 87.55.Qr, 87.56.Fc

## I. INTRODUCTION

For a couple of decades, treatment plans for radiation therapy have been predominantly performed by computer‐based treatment planning systems (TPS). Dose conformity to target volumes while sparing organs at risk is the ultimate goal of using these advanced plans. This goal can be achieved by implementing techniques such as 3D‐conformal radiation therapy, intensity‐modulated radiation therapy (IMRT), and intensity‐modulated arc therapy (IMAT). In combination with these techniques, noncoplanar beams play an important role, especially when the organs at risk need to be better spared without sacrificing the dose distribution conformity of the tumor.^1^, ^2^, ^3^, ^4^, ^5^, ^6^, ^7^, ^8^, ^9^ For example, Wang et al.^(9)^ showed that in 80% of the cases noncoplanar beam configurations were more effective than the coplanar beams to spare the anatomic structures critical to vision for the treatment of paranasal sinus carcinoma. The benefit of using noncoplanar beams was also shown by Laing et al.^(8)^ for the treatment of irregular intracranial targets. Their study indicated that the results of the irregularly shaped target treatment of the static noncoplanar beams were better than those of the multiple‐arc technique. Nevertheless, the preference for the noncoplanar beams will depend on the individual patient case.^9^, ^10^, ^11^ In practice, the use of the noncoplanar beams can be an option for physicians to make adjustments in the treatment plans based on their clinical judgment.

Two conditions have to be fulfilled in order to ensure that patients receive the right dose as prescribed.^(12)^ Firstly, the geometry of the planned beams has to be reproduced accurately by setting up the patient in accordance to the planned position. Secondly, the radiation dose delivered from the machine must be precise. Therefore, verification of the patient positioning and the delivered dose is the key to a successful treatment. Verification of the dose distributions can be achieved using many tools; for example, diodes, thermoluminescent dosimeters, electronic portal imaging devices (EPID), and film.^13^, ^14^, ^15^, ^16^ Although the 2D detector arrays have limited spatial resolution and angular dependency, their applications showed similar results to the 3D dosimeters in terms of the gamma pass rate.^(17)^ Feygelman et al.^(18)^ showed that the 3D diode arrays can also be utilized to reconstruct dose for noncoplanar beams, but only for pretreatment QA. Moreover, due to its advantages for doing online measurements, the application of EPID is extended to validate the dose during patient treatment (*in vivo* dosimetry)^(19)^ by comparing calculated planar dose distributions to the measured ones. The predicted planar dose distributions can be obtained from treatment planning systems (TPS). By creating structures to mimic the measurement equipment (i.e., film or EPID) which are normally perpendicular to the central axis of the beam in the computed tomography (CT) datasets, the planar dose distributions can be calculated. However, with TPS typically available today it is challenging to create this special structure in order to acquire its planar dose from a noncoplanar field. Thus, the noncoplanar fields generated by the TPS are difficult to directly assess utilizing the methods mentioned above.

In the present work, we investigated the feasibility of using in‐house scripts in the Pinnacle^3^ TPS (Philips Radiation Oncology Systems, Fitchburg, WI) to automatically construct structures that mimic the measurement tools and are defined as virtual planes, so that their planar dose distributions of the noncoplanar fields can be calculated. In contrast to the work by Prabhakar et al.,^(20)^ in this study the structures' planar dose for any arbitrary gantry and couch angles, as well as source to planar distance, can be produced to be compared to the measured planar dose. Measurements using two different quality assurance (QA) tools were performed to evaluate the accuracy of the computed noncoplanar fields' planar dose distributions from the scripts. This investigation will potentially lead to future work for verifying the planar dose distribution of noncoplanar fields based on the TPS calculation either as a part of pretreatment QA or in *in vivo* dosimetry.

## II. MATERIALS AND METHODS

### A. Virtual plane in Pinnacle^3^ TPS

**Figure 1:**
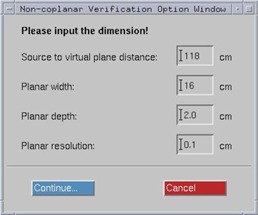
A GUI to create virtual planes.

**Figure 2:**
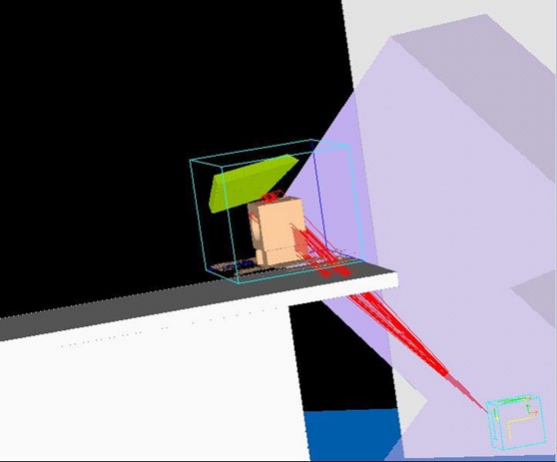
Illustration of a virtual plane created by the scripts for gantry 135° and couch 45°.

Virtual planes or structures to imitate the measurement tools that are perpendicular to the central axis of the beam were generated using in‐house scripts written in the Pinnacle^3^ scripting language. The virtual planes can be positioned for any arbitrary gantry and couch angles to cover both coplanar and noncoplanar fields. The scripts automate the whole process, from creation of the virtual planes, which are based on the configuration of the planned beams, to the calculation of planar dose distributions of all planes. A graphical user interface (GUI) (see Fig. 1) was created that enables a higher flexibility for the user to define the virtual planes' settings. This approach can thus be used to change easily between different measurement devices and/or settings (e.g., film or EPID). Figure 2 shows an example of a created virtual plane that is perpendicular to the gantry at 135° with the couch position at 45°.

### B. Measurements

#### B.1 Measurement setup in Pinnacle^3^ TPS

For validation, virtual planes with the density of water for calculating planar dose distributions were generated in the TPS. Subsequently, the planar dose distributions were verified using two different QA tools, I'm*RT* MatriXX (IBA Dosimetry GmbH, Schwarzenbruck, Germany) and EDR2 film (Carestream Health Inc., Rochester, NY). To include the tissue inhomogeneity effect, a so‐called electron density phantom (EDP) (QRM GmbH, Möhrendorf, Germany), with five tissue equivalent materials as inserts,^(21)^ was utilized. A source to virtual plane distance of 118 cm was selected. The planar dose distributions at depths of 2.33 cm and 2.00 cm were calculated to represent the measurement depths of the I'm*RT* MatriXX and the EDR2 film, respectively. Five couch angles (10°, 20°, 30°, 40°, and 45°) with the same gantry angle (90°) were used as machine settings. In addition, for each couch position, three open fields ($6\times 6\;{\text{cm}}^{2}$, $8\times 8\;{\text{cm}}^{2}$, and $9.8\times 9.8\;{\text{cm}}^{2}$) and one IMRT field with five segments were designed, with a total of 400 MUs applied for every field. The resulting planar dose distributions from the Pinnacle^3^ were multiplied by planned monitor units (MUs), since they were originally in dose fluence units (cGy/MU), to acquire planar absolute dose distributions.

#### B.2 Measurement setup at the treatment delivery system

**Figure 3:**
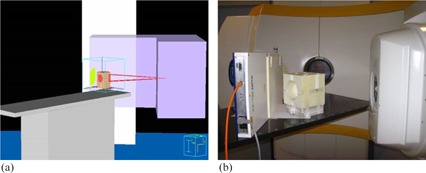
Setup of noncoplanar fields verification: (a) Pinnacle^3^ TPS, (b) measurements at Novalis.

Measurements using 6 MV X‐ray beams from Novalis (BrainLAB, Feldkirchen, Germany) were carried out to verify the planar dose distributions of noncoplanar fields resulting from the scripts written in the Pinnacle^3^ TPS. The I'm*RT* MatriXX and the EDR2 film were placed perpendicular to the beam by means of laser alignment with a setup uncertainty of 1 mm to measure the planar dose distributions as planned in the TPS (see Fig. 3). Moreover, the central line of the EDP phantom was always aligned parallel to the couch. The isocenter of the beams was located at the center of the EDP phantom.

##### B.2.1 Film calibration

For calibration purpose, a film was placed between 30 cm wide ×30 cm long ×1 cm deep RW3 slabs with the configuration of 3 cm above and 5 cm below the film. The film was irradiated using a source‐to‐surface distance (SSD) of 100 cm with the machine gantry angle at 0° that covered a dose range between 30 and 500 cGy. The field size used for this process was $4.2\times 4.2\;{\text{cm}}^{2}$. A calibration curve was obtained from the film using the relation between the image pixel value (PV) and the dose. Instead of using optical density, the PV was taken because it is more sensitive to gray value changes.^(22)^

##### B.2.2 Film dosimetry

EDR2 films from the same batch as for the film calibration were irradiated using the configuration from the TPS with three open fields and one IMRT field, but only at couch angles of 10° and 45°, to represent the extreme condition of couch angles in this study. During the acquisitions, the films were placed between the I'm*RT* MatriXX and 2 cm of RW3, Fig. 3(b). All films were developed at least 12 hrs after irradiation to ensure that the films had reached their stable state.^(23)^ The films were then scanned using a Vidar VXR DosimetryPro 16‐bit scanner (Vidar Systems Corp., Herndon, VA) to obtain the PV.

##### B.2.3 I'mRT MatriXX measurements

All beams from TPS in their treatment orientation were employed to irradiate the MatriXX system. As in the film measurement, 2 cm of RW3 slabs were placed in front of the MatriXX. Furthermore, since the system has 3.3 mm as water‐equivalent absorber thickness, the planar dose distribution from the measurements was compared to the one from the TPS, which was calculated using this geometry.

### C. Data analysis

The films' planar absolute dose distributions of the irradiated noncoplanar fields were made by using the film dose calibration curve, which was created by applying a third order polynomial fit to the measured pixel values of the calibration film. Furthermore, the gamma evaluation method^(24)^ with criteria of 3% delta dose and 3 mm distance to agreement (DTA) was used to evaluate the planar absolute dose maps from the TPS with the measured ones from the films and the MatriXX system. The gamma analysis was performed with the OmniPro I'm*RT* software (IBA Dosimetry GmbH).

## III. RESULTS AND DISCUSSION

**Figure 4:**
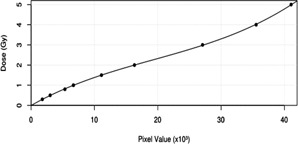
EDR2 film dose calibration curve.

**Figure 5:**
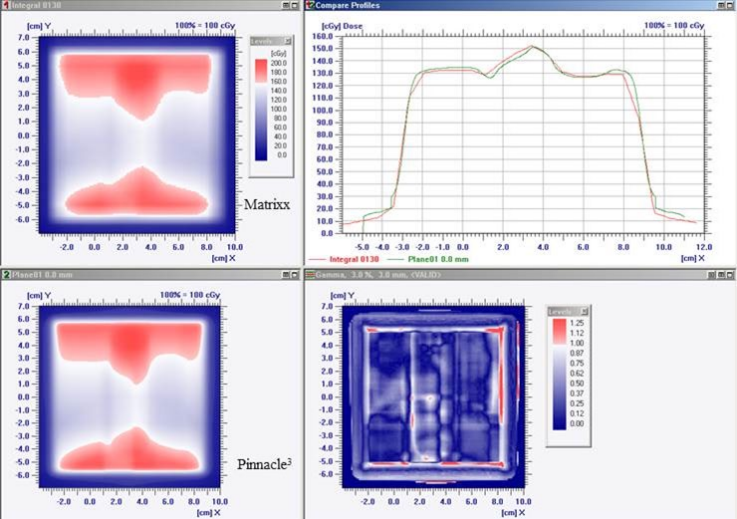
Gamma evaluation of MatriXX and TPS for the $9.8\times 9.8\;{\text{cm}}^{2}$ field and couch angle 45°.

Although the scripts‐generated virtual planes in Pinnacle^3^ can be used to calculate the planar dose for both coplanar and noncoplanar fields, this work particularly focused on the verification of the method in the noncoplanar fields. The planar absolute dose distributions of the irradiated noncoplanar fields using the EDR2 films were determined by employing the calibration curve, shown in Fig. 4. Tables 1 and 2 show that all of the absolute dose patterns measured using both the films and the MatriXX system agree well to the calculated dose distributions at the virtual plane computed by the TPS (γ (3 mm / 3%) pass rate >95%). In addition, the tissue‐inhomogeneity effect from the EDP phantom could be detected in the virtual plane as well (see Fig. 5). Nevertheless, in the IMRT field, the γ (3 mm / 3%) criterion failed in more than 4% of the pixels. This was due to the large dose gradient of the measured dose and from the planning in the border area between each segment.

**Table 1:** Gamma index ≤1 (3% delta dose /3 mm DTA) for absolute dose values of all fields measured by the EDR2 films compared with TPS

	*Couch Angle*	
*Field*	*10°*	*45°*
$6\times 6\;{\text{cm}}^{2}$	97.55%	97.30%
$8\times 8\;{\text{cm}}^{2}$	97.29%	96.97%
$9.8\times 9.8\;{\text{cm}}^{2}$	98.87%	98.64%
IMRT	95.11%	95.14%

**Table 2:** Gamma index ≤1 (3% delta dose /3 mm DTA) for absolute dose values of all fields measured by the MatriXX system compared with TPS

	*Couch Angle*				
*Field*	*10°*	*20°*	*30°*	*40°*	*45°*
$6\times 6\;{\text{cm}}^{2}$	99.67%	99.30%	98.90%	98.30%	97.88%
$8\times 8\;{\text{cm}}^{2}$	96.98%	98.85%	99.20%	99.43%	99.35%
$9.8\times 9.8\;{\text{cm}}^{2}$	97.40%	96.97%	97.27%	96.86%	98.03%
IMRT	96.75%	95.39%	96.69%	96.26%	97.01%

The written Pinnacle^3^ scripts in this work can be used only to generate the virtual planes within the planning CT datasets. Nonetheless, the original CT datasets can be fused to a template CT dataset that contains air only with bigger dimension so that the dimension original CT dataset can be expanded. By using other scripts in Pinnacle^3^, the original plan can be reproduced to the expanded CT. Thus the use of this method can be extended (e.g., for the direct verification (*in vivo* dosimetry) of the noncoplanar field) by using EPID, which is challenging to achieve by utilizing available commercial QA tools.^(18)^ However, this is not part of this study. Furthermore, a new treatment system, Vero, which is a joint product of MHI (Mitsubishi Heavy Industries Ltd., Tokyo, JP) and BrainLAB (BrainLAB AG) can benefit by using the results of this report to validate the planar dose of the noncoplanar fields, since Vero does not have any issue with positioning the EPID behind the patient, as other treatment systems do, because of its O‐ring gantry configuration.

## IV. CONCLUSIONS

In this feasibility study, we have shown that the virtual planes generated by scripts in Pinnacle^3^ can be used to verify the planar dose distributions of the noncoplanar fields. Measurements using two independent QA tools confirmed more than 95% agreement to within 3% of delta dose and 3 mm DTA of the planar absolute dose maps calculated in TPS using the virtual planes. Furthermore, the results of this work can be beneficial either for pretreatment dose verification or for *in vivo* dosimetry that directly assesses the planar dose patterns from the TPS, particularly for the noncoplanar fields.

## ACKNOWLEDGMENTS

The second author performed the presented work in partial fulfilment of the requirements for obtaining the degree of PhD.
